# Thyroid Nodule Detection and Classification on Small Datasets: An Ensemble Deep Learning Approach with Attention Mechanism and Focal Loss

**DOI:** 10.3390/diagnostics16060825

**Published:** 2026-03-10

**Authors:** Wei-Chen Hung, Yi-Kai Chang, Chih-Ming Chang, Po-Wen Cheng, Wu-Chia Lo, Ping-Chia Cheng, Li-Jen Liao

**Affiliations:** 1 Department of Otolaryngology Head and Neck Surgery, Far Eastern Memorial Hospital, New Taipei City 22060, Taiwan; davidhon1990515@gmail.com (W.-C.H.); wishsmile89757@gmail.com (Y.-K.C.); b88401077@ntu.edu.tw (C.-M.C.); powenjapan@yahoo.com.tw (P.-W.C.); lowuchia@gmail.com (W.-C.L.); 2 Department of Communication Engineering, Asia Eastern University of Science and Technology, New Taipei City 22060, Taiwan; 3 Head and Neck Cancer Surveillance and Research Study Group, Far Eastern Memorial Hospital, New Taipei City 22060, Taiwan; 4 Department of Biomedical Engineering, National Yang Ming Chiao Tung University, Taipei 11221, Taiwan; 5 Graduate Institute of Medicine, Yuan Ze University, Taoyuan 32003, Taiwan; 6 Department of Electrical Engineering, Yuan Ze University, Taoyuan 32003, Taiwan

**Keywords:** thyroid nodules, ultrasound imaging, deep learning, ensemble learning, focal loss, attention mechanism, class imbalance

## Abstract

**Background**: Thyroid nodule classification on ultrasound remains challenging due to limited labeled data and marked class imbalance. This study proposes an integrated deep learning framework combining YOLO-based region-of-interest detection with an enhanced ResNet18 classifier. **Methods**: A total of 522 thyroid ultrasound images from 522 patients examined between July 2020 and June 2024 were included. The dataset comprised 467 images for training (399 benign, 68 malignant), 41 for independent testing (19 benign, 22 malignant), and 14 for internal validation (4 benign, 10 malignant). An external validation set of 36 images (22 benign, 14 malignant) was collected from online sources. ResNet18 with a convolutional block attention module was used to enhance feature extraction. To address small sample size and class imbalance, the training pipeline incorporated focal loss, weighted random sampling, mixup augmentation, cosine annealing learning rate scheduling, and a 5-fold cross-validation ensemble. **Results**: The ensemble model achieved 85.4% accuracy (95% CI: 74.5–96.2%), 86.4% sensitivity (95% CI: 72.0–100%), and 84.2% specificity (95% CI: 67.8–100%) on the independent test set. Internal validation yielded 85.7% accuracy, 90.0% sensitivity, and 75.0% specificity, while external validation demonstrated 77.8% accuracy, 78.6% sensitivity, and 77.3% specificity. These findings suggest that advanced regularization combined with ensemble learning improves generalizability despite limited data. **Conclusions**: This study demonstrates that a lightweight ResNet18 architecture with strategic optimization outperforms deeper networks on small medical datasets. The proposed framework demonstrated good diagnostic performance across multiple validation cohorts, offering a promising computer-aided diagnosis tool for thyroid nodule assessment.

## 1. Introduction

Thyroid nodules are highly prevalent, appearing in up to 60% of the general population via high-resolution ultrasound [1,2]. Despite their high prevalence, only 5–15% of nodules are malignant [3]. The clinical challenge is to accurately identify this malignant minority to guide interventions such as fine-needle aspiration (FNA), while minimizing unnecessary procedures for benign nodules. To standardize ultrasound-based risk stratification, the American College of Radiology (ACR) proposed the Thyroid Imaging Reporting and Data System (TI-RADS), which scores nodules according to composition, echogenicity, shape, margins, and echogenic foci [4]. However, interpreting these sonographic features remains subjective. Distinguishing microcalcifications from benign colloid crystals may be challenging for less experienced clinicians [5].

Before the rise of deep learning (DL), researchers sought to mitigate subjectivity through radiomics. This workflow extracted handcrafted features to quantify texture (such as Gray Level Co-occurrence Matrix), shape, and signal intensity. These quantitative features were then processed by machine learning (ML) classifiers, such as Support Vector Machines (SVMs) and Random Forests (RFs). While these methods demonstrated high diagnostic accuracy in controlled settings [6], they are inherently limited by their reliance on predefined features, which often fail to capture the complex, high-level patterns present in medical imaging [7].

The advent of DL, particularly Convolutional Neural Networks (CNNs), has addressed these limitations by enabling the automated learning of hierarchical representations from images [8,9]. Recent studies have successfully applied transfer learning from natural image datasets (e.g., ImageNet) to thyroid ultrasound, with architectures like GoogLeNet and ResNet-50 achieving performance metrics comparable to expert radiologists [10,11,12].

Despite the rapid evolution, significant barriers persist in transitioning these models to clinical practice. First, unlike natural image benchmarks such as ImageNet, which provide millions of annotated samples, medical datasets are typically small and suffer from severe class imbalance due to the low natural incidence of malignancy [13,14]. Under such class imbalance, conventional models can achieve high accuracy by simply predicting the dominant benign class, rendering them clinically useless for effective screening [15]. Second, most of current literature relies on manually cropped images for classification tasks [16,17]. This approach bypasses the initial step of nodule localization, creating a bottleneck in fully automated clinical workflows. The development of integrated pipelines that seamlessly perform both automated detection and classification remains comparatively underexplored.

To address these gaps, we propose an end-to-end pipeline that integrates object detection and classification, effectively mirroring the detect-then-diagnose workflow of a radiologist. Our framework incorporates specialized optimization strategies, including focal loss [18,19], weighted random sampling [20], mixup augmentation [21], cosine annealing learning rate scheduling [22], and a 5-fold cross-validation ensemble [12], to enhance sensitivity toward the minority malignant class. By evaluating our approach on both internal and external validation cohorts, we aim to demonstrate a robust and generalizable tool for high-sensitivity thyroid nodule screening.

## 2. Materials and Methods

### 2.1. Patient Selection and Data Preparation

**Figure 1:**
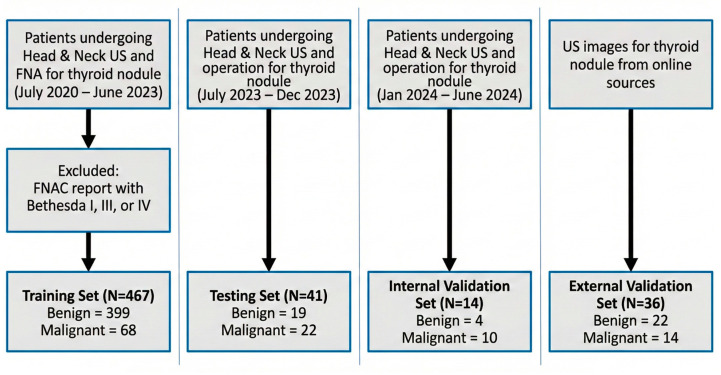
Flowchart depicting the study’s inclusion and exclusion criteria. Abbreviation: US, ultrasound.

This retrospective study was conducted at a single medical center, in accordance with the principles of the Declaration of Helsinki, and was approved by the institutional review board (IRB No. 113273-E). Patient selection and the corresponding inclusion and exclusion criteria are summarized in Figure 1.

Training/validation set: We retrospectively screened patients who underwent head and neck ultrasound and fine-needle aspiration (FNA) for thyroid nodules between July 2020 and June 2023. To ensure diagnostic certainty, we only included cases with definitive pathological or cytological outcomes (Bethesda categories II, V, or VI). Patients presenting with non-diagnostic or indeterminate cytology (Bethesda I, III, or IV) were excluded to ensure diagnostic certainty. The reference standard was defined by surgical pathology where available; otherwise, cytological results were used (Bethesda II as benign; Bethesda V/VI as malignant).

Independent testing set: To provide a robust and unbiased evaluation of the model, an independent temporal testing set was established. This cohort included consecutive patients who underwent thyroid ultrasound followed by surgical resection between July 2023 and December 2023. Relying exclusively on surgical pathology for this testing set ensures a highly reliable, histopathology-confirmed ground truth for assessing diagnostic performance.

All head and neck ultrasound examinations were performed by two otolaryngologists with more than 10 years of experience, using a Toshiba Aplio 500 system (Canon Medical Systems, Tochigi-ken, Japan) equipped with a 5–14 MHz linear-array transducer. For each patient, a single representative ultrasound image was selected, corresponding to the plane showing the transverse axis. Images were retrieved from the institutional Picture Archiving and Communication System (PACS). All identifiable information, including name, date of birth, admission date, and medical record number, was removed using the Microsoft Snipping Tool to ensure patient confidentiality. Clinical data, including age, gender, tumor side, tumor size, and corresponding cytology and/or pathology results, were analyzed and compared between the training/validation set and the independent testing set.

### 2.2. Image Preprocessing and ROI Detection

Image preprocessing consisted of two main steps. First, region-of-interest (ROI) detection was performed using YOLOv8, a state-of-the-art object detection model, to automatically localize and crop the nodule from the full ultrasound image. All images in the training and testing sets were manually annotated with bounding boxes by two experienced otolaryngologists using Labelimg. Inter-observer discrepancies were resolved through consensus discussion. YOLOv8 was initially trained on the training set, achieving a precision of 0.969, recall of 0.932, and mAP50 of 0.984 on the independent testing set (using the YOLOv8n variant with 640 × 640-pixel input images). All generated ROIs during validation were visually reviewed by a third otolaryngologist to confirm anatomic correctness, with no systematic errors identified. A 2.5% margin was added around the predicted bounding box to preserve the nodule–tissue interface, including features such as irregular margins, while excluding surrounding thyroid tissue, muscle, and textual overlays. Second, the cropped ROIs were resized to 224 × 224 pixels and normalized using ImageNet statistics (mean = [0.485, 0.456, 0.406], std = [0.229, 0.224, 0.225]) to facilitate transfer learning with pretrained convolutional backbones.

### 2.3. Final Network Architecture: ResNet18 + CBAM

**Figure 2:**
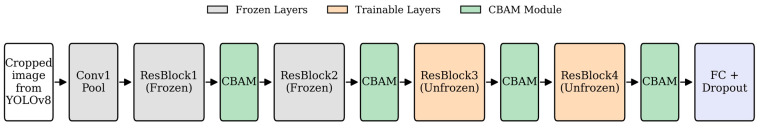
Schematic illustration of the proposed ResNet18-CBAM architecture. Abbreviations: CBAM, Convolutional Block Attention Module; FC, Fully Connected layer; GAP, Global Average Pooling; ResNet, Residual Network.

Our preliminary experiments revealed that deeper architectures, such as ResNet-50, were prone to overfitting on this dataset, while freezing pre-trained weights led to underfitting. Therefore, we selected ResNet18 as the optimal backbone. Its residual learning framework facilitates stable optimization in deeper networks, while its relatively concise depth (compared with ResNet50 or ResNet101) provides an implicit structural regularization that helps limit overfitting on small datasets. To further balance model stability and adaptability, we employed a gradual unfreezing fine-tuning protocol. To further enhance feature extraction, the Convolutional Block Attention Module (CBAM) was inserted after each residual block (Figure 2). CBAM sequentially applies two complementary attention mechanisms [23]: (1) channel attention, which emphasizes the most informative feature channels (such as calcification-related textures), and (2) spatial attention, which focuses on the most relevant regions within the feature map (such as irregular nodule margins). The attention refinement can be expressed as:

(1) F′ = Mc(F) ^⊗^ F

(2) F″ = Ms(F′) ^⊗^ F′

where F denotes the input feature map, Mc and Ms denote the channel and spatial attention modules, respectively, and ^⊗^ indicates element-wise multiplication.

### 2.4. Loss Function

We employed Focal Loss (FL) to overcome the inherent class imbalance in thyroid ultrasound images. Standard Cross-Entropy (CE) loss is often dominated by easily classified majority examples, which limits the model’s sensitivity to the minority class. To address this, FL reshapes the conventional cross-entropy loss by down-weighting well-classified examples, forcing the network to focus on harder and misclassified examples [18]. The FL in Equation (3) is defined as:

(3) $$FL\left(p_{t}\right)=-\alpha_{t}{\left(1-p_{t}\right)}^{\gamma }log(p_{t})$$

where $p_{t}$ is the model’s estimated probability for the ground-truth class. The modulating factor ${\left(1-p_{t}\right)}^{\gamma }$ reduces the loss contribution from easy examples (when $p_{t}$ → 1), with the focusing parameter γ controlling the strength of down-weighting effect. In this study, the balancing parameter *α*_t_ was set to 1 and *γ* was set to 2. This objective function has been shown to stabilize training and improve classification performance in imbalanced medical ultrasound datasets [19]

### 2.5. Class Balancing

To further counter class imbalance, we employed a weighted random sampling strategy during the training phase. This method assigns a higher sampling probability to the minority class (malignant nodules), ensuring that each mini-batch maintains a balanced distribution of classes [20].

### 2.6. Data Augmentation

We utilized Mixup augmentation Equation (4) to enhance model generalization and robustness against ultrasound artifacts [21]. By generating synthetic training examples through combinations of image pairs and their labels, Mixup encourages the model to behave linearly between training examples [24]. A virtual training example is generated as:

(4) x′ = λxi + (1 − λ)xj and y′ = λyi + (1 − λ)yj

where λ is drawn from a Beta(α,α) distribution with α = 0.2. Additional augmentations included random horizontal flips, rotations (±10°), and brightness/contrast adjustments (±10%).

### 2.7. Optimizer and Learning Rate Scheduling

To optimize the training process, we used the Adam optimizer (initial learning rate = 1 × 10^−4^, weight decay = 1 × 10^−2^) and a Cosine Annealing Learning Rate [22]. The learning rate *η_t_* Equation (5) is modulated as:

(5) η_t_ = η_min_ + 0.5(η_max_ − η_min_)(1 + cos(πT_cur_/T_i_))

This strategy decays the learning rate smoothly from *η*_max_ to *η*_min_, periodically resetting it to allow the model to escape sharp local minima and converge to a flatter, more generalizable solution.

### 2.8. Ensemble Inference

To mitigate the variance associated with random data splitting and to maximize the utilization of the limited dataset, a 5-fold stratified cross-validation ensemble was used by splitting the dataset into five stratified folds and training separate models on each. During inference, predicted probabilities were averaged (soft voting) to produce the final diagnosis, a method proven to improve reliability [12].

### 2.9. Statistical Analysis

Diagnostic performance was evaluated using accuracy, sensitivity, and specificity, derived from the confusion matrix across the training, validation, and testing sets. Sensitivity and specificity specifically reflect the model’s ability to identify a malignant thyroid nodule.

### 2.10. Internal and External Validation

To rigorously evaluate clinical applicability, two distinct validation sets were established. The Internal Validation Set consisted of 14 images (4 benign, 10 malignant) from patients meeting the study inclusion criteria who underwent ultrasound and surgery between January 2024 and June 2024. The External Validation Set comprised 36 images (22 benign, 14 malignant) obtained from a public repository (Ultrasound Cases, https://www.ultrasoundcases.info, accessed on 26 November 2025) with usage permission. This external cohort was included to assess the model’s generalizability across different imaging equipment and acquisition protocols.

## 3. Results

A total of 558 thyroid ultrasound images were included in this study. The internal cohort consisted of 522 patients examined between July 2020 and June 2024, divided into a training/validation set (*n* = 467; 399 benign, 68 malignant), an independent testing set (*n* = 41; 19 benign, 22 malignant), and an internal validation set (*n* = 14; 4 benign, 10 malignant). Additionally, an external validation set comprising 36 images (22 benign, 14 malignant) was obtained from online sources.

Age, gender distribution, tumor side, and nodule size (short and long axis) were comparable between the two cohorts, with no statistically significant differences (all *p* > 0.05, Table 1). As expected, diagnostic confirmation and final diagnosis differed. Most training cases were confirmed by fine-needle aspiration, whereas all testing cases had surgical pathology, leading to a higher proportion of malignant nodules in the test set (53.7% vs. 14.6%, *p* < 0.001). Detailed information was attached in the Supplementary Materials.

Table 2 details the iterative seven experiments. Initial experiments using a ResNet50 backbone with weighted sampling achieved 100% training accuracy but demonstrated poor generalization, with a test accuracy of 51.2% and a critically low sensitivity of 13.6%, indicating severe overfitting. Subsequent experiments employing ResNet18 with CBAM and early stopping improved training stability; however, performance remained inconsistent with fluctuating accuracy. The freezing strategy (Experiment 4) further reduced test sensitivity to 40.9%, suggesting limited adaptability of partially frozen pretrained features to ultrasound data. Incorporating Mixup augmentation and label smoothing (Experiment 6) improved test accuracy to 75.6% and sensitivity to 68.2%, yet signs of overfitting persisted. Ultimately, the ResNet18 model augmented with CBAM and incorporating focal loss, weighted sampling, Mixup augmentation, and cosine annealing within a 5-fold ensemble framework (Experiment 7) achieved the most balanced performance, demonstrating high sensitivity and stable generalization. The complete training configuration, optimized for model robustness and generalization, is presented in Table 3, while the layer-by-layer architecture is detailed in Table 4. Early stopping based on peak validation F1 scores ensured stability and prevented overfitting. All folds converged robustly: Fold 0 halted at epoch 33 (F1: 0.7224), Fold 1 at epoch 41 (F1: 0.7588), Fold 2 at epoch 45 (F1: 0.7057), Fold 3 at epoch 24 (F1: 0.8319), and Fold 4 at epoch 49 (F1: 0.7584). These results confirm the architecture’s reliability across data splits.

**Figure 3:**
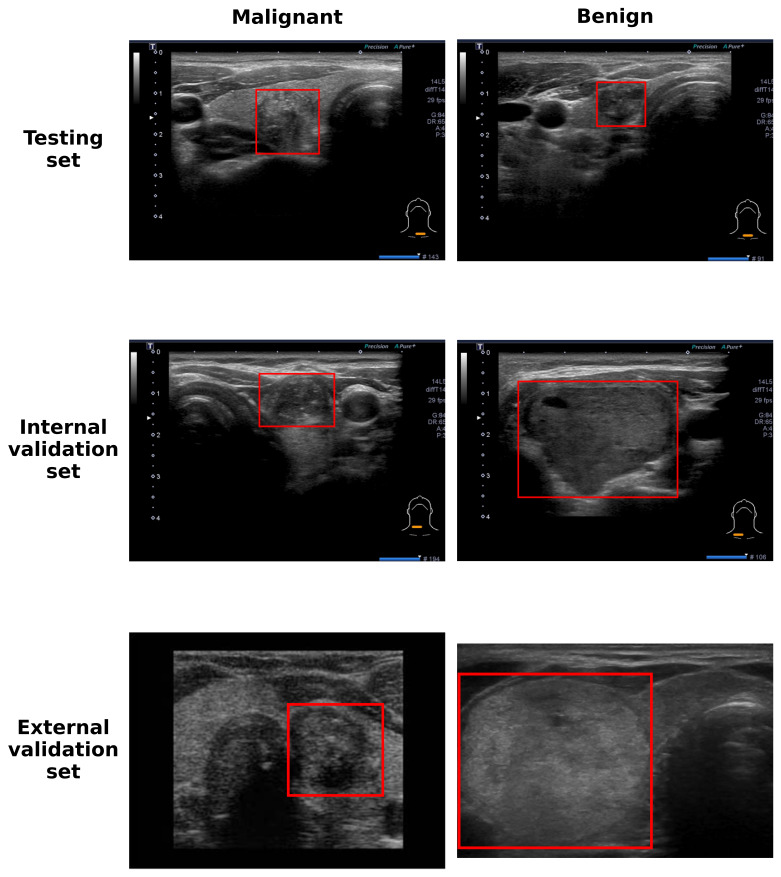
Examples of predicted bounding box across testing, internal validation, and external validation sets. The red bounding box correctly identified the tumor region.

To facilitate efficient deployment, the final 5-fold ensemble model was converted to half-precision floating-point (FP16) format, effectively reducing the model size without compromising accuracy. The complete end-to-end pipeline integrates automated detection with classification: raw ultrasound images are first processed by YOLOv8 to generate cropped ROIs (incorporating a 2.5% margin), which are subsequently resized to 224 × 224 pixels and analyzed by the ResNet18–CBAM ensemble. Figure 3 illustrates examples of predicted bounding boxes across testing, internal validation, and external validation sets. This integrated system demonstrated robust diagnostic performance across all validation cohorts, as detailed in Table 5. On the independent testing set, the model achieved an accuracy of 85.4% a sensitivity of 86.4%, and a specificity of 84.2%. Performance remained consistent in the internal validation set, with an accuracy of 85.7% and a high sensitivity of 90.0%. The imbalanced sensitivity and specificity were probably due to the very small sample size of benign cases (*n* = 4) in this subset. Furthermore, in the external validation set sourced from different equipment and protocols, the model maintained an accuracy of 77.8%, with a sensitivity of 78.6% and specificity of 77.3%. These results indicate that the proposed pipeline effectively mitigates class imbalance and generalizes well to unseen data.

## 4. Discussion

The primary objective of this study was to develop a robust, end-to-end deep learning framework capable of accurately classifying thyroid nodules despite limited data and severe class imbalance. Our proposed pipeline, integrating YOLOv8-based detection with a ResNet18-CBAM classifier, demonstrated clinically meaningful performance. On the independent testing set (Table 5), the model achieved an accuracy of 85.4%, a sensitivity of 86.4%, and a specificity of 84.2%. Notably, the system maintained an accuracy of 77.8% and a sensitivity of 78.6% on an external validation cohort acquired from a different source. These findings suggest that the learned features generalize across different patient populations and imaging protocols, effectively mitigating the domain shift often encountered in ultrasound analysis.

A key finding from our experiments is that increasing model depth does not inherently improve generalization in the medical ultrasound domain (Table 2). The deeper ResNet50 architecture achieved near-perfect performance on the training set but suffered a dramatic drop in sensitivity on the test set, indicating severe overfitting. This reflects the over-parameterization phenomenon, where high-capacity models memorize noise rather than learning generalizable features, particularly when training data is limited [25]. In contrast, the relatively shallow ResNet18 backbone, when combined with structural and algorithmic regularization, demonstrated more stable performance on our small dataset.

The progressive improvement observed across experiments also highlights the importance of explicitly addressing class imbalance (Table 2). Early models relying solely on weighted sampling or early stopping struggled to balance the trade-off between sensitivity and specificity. The integration of Focal Loss was pivotal. By down-weighting easy negatives (benign nodules), the network was compelled to focus on hard, minority-class examples. When combined with weighted random sampling to maintain balanced mini-batches and Mixup augmentation to linearize decision boundaries, this approach shifted the optimization landscape toward improved malignancy detection. Additionally, the use of Cosine Annealing stabilized training by helping the network escape sharp local minima, further enhancing generalization.

Implementing a 5-fold ensemble strategy was essential for mitigating the high variance often observed in small datasets. While single-model configurations showed inconsistent performance across folds, the soft-voting ensemble smoothed these fluctuations, reducing the impact of random initialization and data-splitting bias. The external validation results, although slightly lower than internal metrics (Accuracy 77.8% vs. 85.7%), remain encouraging. Such performance degradation aligns with known challenges in medical image analysis, where domain shifts commonly introduce a generalization gap [26]. Notably, the preserved sensitivity demonstrates the robustness of our pipeline to variations in acquisition equipment, supporting its potential as an adjuvant diagnostic tool in settings with limited subspecialty expertise.

Table 6 compares our pipeline with recent small-sample (<1000 cases) thyroid ultrasound studies that integrate both detection and classification. Developing end-to-end frameworks on limited medical datasets remains challenging, often requiring specialized strategies to ensure robust performance. For instance, Abdelrazik et al. combined U-Net and ResNet-18 on 349 images, using segmentation masks (Dice score: 0.78) to reduce noise prior to classification, but achieved only 78% accuracy [27]. Similarly, Vahdati et al. paired YOLOv5 with XGBoost on 983 patients, incorporating transverse and longitudinal views to reach mAP50 of 0.79 and sensitivity of 84%, though specificity was lower at 63% [28]. In comparison, our YOLOv8 + ResNet18-CBAM pipeline on 522 images achieved superior and balanced performance: mAP50 of 0.98 for detection, and classification accuracy of 85% with sensitivity 86% and specificity 84%. This improvement highlights the synergistic effect of our integrated approach. By utilizing CBAM to emphasize nodule features, Focal Loss to manage class imbalance, and ensemble learning to stabilize training, the framework successfully mitigates the overfitting and generalization issues typical of small medical datasets.

Several clinical and research implications emerge from these findings. First, combining automated nodule localization with a lightweight, attention-enhanced classifier effectively mimics the radiologist’s workflow of detection followed by diagnosis, rather than relying on full-frame classification. Second, our results emphasize that careful loss design, sampling strategies, and ensembling can have a greater impact than simply increasing model depth in ultrasound applications. This framework could serve as an AI assistant during ultrasound reporting, offering benign versus malignant assessments as a second opinion to the TI-RADS score. It has the potential to reduce inter-observer variability, particularly among less experienced clinicians, while prompting careful evaluation of sonographic features for AI-flagged malignancies.

The present study has several limitations. First, the sample size was relatively small, limiting statistical power and resulting in wider confidence intervals for performance metrics. Additionally, retrospective data collection over several years risked minor inconsistencies, though this was mitigated by two highly experienced otolaryngologists performing all head and neck ultrasounds using a single machine. Second, the independent testing set consisted exclusively of surgically confirmed cases, leading to a higher malignancy rate (53.7%) compared to the training set (14.6%). While this ensures rigorous ground truth labeling, it introduces a selection bias that differs from the general screening population, potentially affecting positive predictive value in real-world deployment. Third, we analyzed only B-mode grayscale images. Although Color Doppler aids vascularity assessment, we excluded it to improve model generalizability across ultrasound systems. We also did not map predictions to ACR TI-RADS categories, limiting direct comparisons with subjective clinical scoring. Finally, this was a single-center retrospective study, which may limit generalizability to other scanners and clinical settings. Prospective trials are necessary to evaluate whether this AI-assisted workflow translates to improved clinical decision-making.

## 5. Conclusions

This study demonstrates that a lightweight, strategically optimized deep learning framework can outperform deeper architectures for thyroid nodule classification on small and imbalanced datasets. By integrating YOLOv8 for automated ROI detection and a ResNet18-CBAM classifier, the proposed end-to-end pipeline achieved an accuracy of 85.4%, sensitivity of 86.4%, and specificity of 84.2% on the independent test set, with comparable performance on internal and external validation cohorts. Incorporating focal loss, weighted sampling, Mixup, and cosine annealing within a 5-fold ensemble effectively mitigated class imbalance and overfitting. Additionally, optimizing the ResNet18-CBAM classifier to FP16 precision ensures computational efficiency, facilitating clinical deployment. These results suggest that the proposed framework represents a promising and generalizable computer-aided diagnosis tool to assist clinicians in accurate thyroid risk stratification.

## Figures and Tables

**Table 1:** Comparison of clinical characteristics between training/validation and testing sets.

Clinical Characteristics, Mean (SD) or *N* (%)	Train/Val	Test	*p* Value
	*N* = 467	*N* = 41	
Age, year	51.27 (12.47)	49.29 (10.94)	0.326
Gender			0.862
Female	321 (68.7%)	29 (70.7%)	
Male	146 (31.3%)	12 (29.3%)	
Tumor side			0.190
Left	207 (44.3%)	23 (56.1%)	
Right	260 (55.7%)	18 (43.9%)	
Tumor size			
Short axis, cm	1.52 (0.80)	1.65 (0.85)	0.3419
Long axis, cm	2.17 (1.19)	2.47 (1.62)	0.1412
Diagnostic confirmation			<0.001 *
Fine-needle aspiration	351 (75.2%)	0 (0.0%)	
Operation	116 (24.8%)	41 (100.0%)	
Final diagnoses			<0.001 *
Benign tumors	399 (85.4%)	19 (46.3%)	
Malignant tumors	68 (14.6%)	22 (53.7%)	

Note: Statistical significance with *p* < 0.05 is highlighted with an asterisk.

**Table 2:** Summary of model architectures and outcomes for each experiment.

No	Architecture	Strategy	Dataset	Accuracy	Sensitivity	Specificity	Interpretation
1	ResNet50	Weighted sampler	Train	100.0%	100.0%	100.0%	The model exhibited overfitting and toward the benign class.
			Val	88.3%	28.6%	98.8%	
			Test	51.2%	13.6%	94.7%	
2	ResNet18	Downsizing + data augmentation + dropout	Train	80.4%	90.3%	69.5%	The model suffered from overfitting with inconsistent accuracy
			Val	62.8%	64.3%	62.5%	
			Test	85.4%	86.4%	84.2%	
3	ResNet18 + CBAM	Early stopping	Train	100.0%	100.0%	100.0%	The model kept suffering from overfitting.
			Val	88.3%	21.4%	100.0%	
			Test	68.3%	45.5%	94.7%	
4	ResNet18 (Freezing) + CBAM	Early stopping + freezing + use f1-score as metrics	Train	67.8%	77.2%	58.7%	The model exhibited underfitting and poor generalization
			Val	80.9%	64.3%	83.8%	
			Test	65.9%	40.9%	94.7%	
5	ResNet18 (Fine-Tuning) + CBAM	Fine-tuning (unfreeze l3/4)	Train	97.8%	100.0%	95.4%	Signs of overfitting reemerged
			Val	80.8%	35.7%	88.8%	
			Test	71.0%	59.0%	84.0%	
6	ResNet18 (Regularization)	Mixup + label smoothing	Train	96.7%	100.0%	93.7%	Mixup slightly mitigated overfitting
			Val	80.9%	42.9%	87.5%	
			Test	75.6%	68.2%	84.2%	
7 *	ResNet18 (K-Fold Ensemble) + CBAM	Focal loss + cosine annealing	Train	85.0%	100.0%	82.4%	Focal loss prioritizes malignancy, achieving high sensitivity and stable results
			Val	88.6%	100.0%	86.6%	
			Test	85.4%	86.4%	84.2%	

* The final selected model is marked with an asterisk.

**Table 3:** Proposed ensemble model configuration summary.

Component	Specification	Purpose
**Backbone**	ResNet-18	Feature extraction (Prevent overfitting on small data)
**Attention Module**	CBAM (Channel + Spatial)	Focus on nodule margins and heterogeneous texture
**Loss Function**	Focal Loss (alpha = 1, gamma = 2)	Address class imbalance
**Optimizer**	Adam (LR = 1 × 10^−4^)	Better regularization with weight decay
**Scheduler**	Cosine Annealing	Escape local minima and improve generalization
**Sampling**	Weighted Random Sampler	Ensure 1:1 class ratio in training batches
**Augmentation**	Mixup	Increase data diversity and robustness
**Inference**	5-Fold Ensemble (Soft Voting)	Reduce variance and improve stability

Abbreviation: CBAM, Convolutional Block Attention Module.

**Table 4:** Architecture of the ResNet18-CBAM Model.

Layer Name	Kernel	Output Size (H × W × C)	Remarks
Input Image	YOLOv8 bbox detection	224 × 224 × 3	Cropping and then resizing
Conv1	7 × 7, 64, stride 2	112 × 112 × 64	Frozen (Pre-trained)
MaxPool	3 × 3, stride 2	56 × 56 × 64	Frozen (Pre-trained)
Layer 1 + CBAM1	[3 × 3, 64; 3 × 3, 64] × 2 + CBAM	56 × 56 × 64	Frozen; Includes CBAM
Layer 2 + CBAM2	[3 × 3, 128; 3 × 3, 128] × 2 + CBAM	28 × 28 × 128	Frozen; Includes CBAM
Layer 3 + CBAM3	[3 × 3, 256; 3 × 3, 256] × 2 + CBAM	14 × 14 × 256	Unfrozen for fine-tuning; Includes CBAM
Layer 4 + CBAM4	[3 × 3, 512; 3 × 3, 512] × 2 + CBAM	7 × 7 × 512	Unfrozen for fine-tuning; Includes CBAM
Global Avg Pool	Adaptive Average Pooling	1 × 1 × 512	Feature aggregation
Classification Head	Dropout (0.5) + Linear (512, 2)	1 × 2	Binary output: Benign vs. Malignant

Abbreviation: CBAM, Convolutional Block Attention Module; H × W × C, Height × Width × Channels; ResNet, Residual Network; YOLO, You Only Look Once.

**Table 5:** Performance Metrics of the Proposed Ensemble Model on various datasets.

Data Set	TP	FN	FP	TN	ACC	SEN	SPE
Testing	19	3	3	16	85.4% (74.5–96.2%)	86.4% (72.0–100%)	84.2% (67.8–100%)
Internal validation	9	1	1	3	85.7% (67.4–100%)	90.0% (71.4–100%)	75.0% (32.6–100%)
External validation	11	3	5	17	77.8% (64.2–91.4%)	78.6% (57.1–100%)	77.3% (59.8–94.8%)

Abbreviations: TP, true positive; FN, false negative; FP, false positive; TN, true negative; ACC, accuracy; SEN, sensitivity; SPE, specificity.

**Table 6:** Small-sample (<1000 cases) thyroid ultrasound studies in the past five years (including both detection and classification tasks).

Reference	Architecture	Dataset	Detection	Classification	Key Strategy
Abdelrazik et al. (2025) [27]	U-Net + ResNet-18	349 images	IoU: 0.68 Dice: 0.78	ACC: 78%	Removal of background noise via segmentation masks prior to classification
Vahdati et al. (2024) [28]	YOLOv5 + XGBoost	983 patients	mAP 50: 0.79	AUC: 0.84 SEN: 84% SPE: 63%	Integrating both transverse and longitudinal views
Our Study (Proposed Model)	YOLOv8 + ResNet18-CBAM	522 images	mAP50: 0.98	ACC: 85.4% SEN: 86.4% SPE: 84.2%	Integrated CBAM attention, Focal Loss, and Ensemble learning.

Abbreviation: ACC, Accuracy; AUC, Area under the receiver operating characteristic curve; Dice, Dice similarity coefficient; IoU, Intersection over Union; mAP50, Mean Average Precision at an IoU threshold of 0.5; SEN, Sensitivity; SPE, Specificity; U-Net, U-shaped Convolutional Neural Network; XGBoost, Extreme Gradient Boosting; YOLO, You Only Look Once; CBAM, Convolutional Block Attention Module.

## Data Availability

The original contributions presented in this study are included in the article. Further inquiries can be directed to the corresponding authors.

## Supplementary Materials

The following supporting information can be downloaded at: https://www.mdpi.com/article/10.3390/diagnostics16060825/s1. The data set is available in the Supplementary Table S1: Detailed clinical characteristics of the included patients.

## Abbreviations

The following abbreviations are used in this manuscript:

ACC	Accuracy
AUC	Area under the receiver operating characteristic curve
ACR	American College of Radiology
CBAM	Convolutional Block Attention Module
CE	Cross-Entropy
CI	Confidence Interval
CNNs	Convolutional Neural Networks
DL	Deep Learning
FC	Fully Connected layer
FL	Focal Loss
FN	False Negative
FNA	Fine-Needle Aspiration
FP	False Positive
FP16	Half-precision floating-point
GAP	Global Average Pooling
IoU	Intersection over Union
LR	Learning Rate
mAP50	Mean Average Precision at an IoU threshold of 0.5
ML	Machine Learning
PACS	Picture Archiving and Communication System
ResNet	Residual Network
RFs	Random Forests
ROI	Region-of-Interest
SD	Standard Deviation
SEN	Sensitivity
SPE	Specificity
SVMs	Support Vector Machines
TI-RADS	Thyroid Imaging Reporting and Data System
TN	True Negative
TP	True Positive
US	Ultrasound
XGBoost	Extreme Gradient Boosting
YOLO	You Only Look Once
